# Up-regulation of ATP-binding cassette transporters in the THP-1 human macrophage cell line by the antichagasic benznidazole

**DOI:** 10.1590/0074-02760160080

**Published:** 2016-10-24

**Authors:** Virginia G Perdomo, Juan P Rigalli, Marcelo G Luquita, José M Pellegrino, María Laura Ruiz, Viviana A Catania

**Affiliations:** 1 Instituto de Fisiología Experimental, Facultad de Ciencias Bioquímicas y Farmacéuticas, Rosario, Argentina; 2 University of Heidelberg, Department of Clinical Pharmacology and Pharmacoepidemiology, Heidelberg, Germany

**Keywords:** ABC transporters, benznidazole, macrophage cells

## Abstract

The effect of benznidazole (BZL) on the expression and activity of P-glycoprotein (P-gp, ABCB1) and multidrug resistance-associated protein 2 (MRP2, ABCC2), the two major transporters of endogenous and exogenous compounds, was evaluated in differentiated THP-1 cells. BZL induced P-gp and MRP2 proteins in a concentration-dependent manner. The increase in mRNA levels of both transporters suggests transcriptional regulation. P-gp and MRP2 activities correlated with increased protein levels. BZL intracellular accumulation was significantly lower in BZL-pre-treated cells than in control cells. PSC833 (a P-gp inhibitor) increased the intracellular BZL concentration in both pre-treated and control cells, confirming P-gp participation in BZL efflux.

Treatment of intracellular infections requires the use of chemotherapeutic agents capable of penetrating eukaryotic cells and reaching an optimal concentration to eliminate the causal agent (Tulkens 1990). The intracellular concentration of a given drug results from influx and efflux processes that can determine its chemotherapeutic efficacy and toxicity. P-glycoprotein (P-gp, MDR1, ABCB1) and multidrug resistance-associated protein 2 (MRP2, ABCC2) are recognised members of the ATP-binding cassette (ABC) superfamily of efflux proteins, localised mainly in the plasma membrane of many cell types. Most of therapeutic agents used to treat intracellular infections are substrates of these transporters (Köhle & Bock 2009, Klaassen & Aleksunes 2010).

Benznidazole (BZL) is the only drug that is available in most endemic countries for the treatment of Chagas disease or American trypanosomiasis, a neglected illness that affects approximately 6 million people in endemic areas; in addition, another 70 million people are at risk of infection (Coura & de Castro 2002, WHO 2015). The occurrence of this zoonosis in non-endemic areas, such as the United States and Europe, is mainly due to the migration of infected people from endemic areas (Gascón et al. 2010). The etiological agent of Chagas disease is the parasite *Trypanosoma cruzi*, a haemoflagellate protozoan that is predominantly transmitted to humans by haematophagous insect vectors. In addition, *T. cruzi* can be transmitted by transfusion of infected blood, ingestion of contaminated food, vertically from mother to infant, organ transplantation, and laboratory accidents. The life cycle of *T. cruzi* is complex, with several developmental stages in insect vectors and mammalian hosts including epimastigotes (insect-replicative form), trypomastigotes (infective and non-replicative form in mammalian or insect host), and amastigotes (infective and intracellular replicative form in mammalian host). Once trypomastigotes enter the dermis or conjunctival membrane, all types of nucleated cells in the human host are potential targets of infection. Since the parasite invades a variety of cell types at the inoculum site, and since macrophages are a type of cell in which initial *T. cruzi* replication occurs, these cells represent a site where optimal intracellular BZL concentration is required (Brener 1980 , Coura & Borges-Pereira 2010).

BZL efficacy is variable and until now, the reasons for this have been unclear. Different drug susceptibilities of *T. cruzi* strains, induction of drug resistance in the parasite during treatment, or limited tissue penetration have been postulated to alter BZL efficacy (Murta et al. 1998, Coura & de Castro 2002, Urbina & Docampo 2003, d, dos Santos et al. 2008, Nogueira et al. 2012).

In previous studies, we have observed the induction of P-gp and MRP2/Mrp2 by BZL in HepG2 cells (a cell model of human hepatocytes) (Rigalli et al. 2012) and in the liver and intestine of BZL-treated rats (Perdomo et al. 2013). In addition, we described that P-gp is involved in BZL extrusion (Rigalli et al. 2012) and subsequently, modifications in BZL pharmacokinetics in rats due to the induction of P-gp were observed (Perdomo et al. 2013). It was demonstrated that changes in ABC transporter activities could affect the therapeutic efficacy of drugs (Jorajuria et al. 2004, Bellusci et al. 2013). If macrophages exhibit similar ABC induction during BZL treatment, therapeutic failure might be expected.

Until now, no information has been available regarding the effect of BZL on efflux transporters in macrophages. Thus, the aim of the present study was to evaluate the effect of BZL pre-treatment on the expression and activity of P-gp and MRP2, the two major transporters of endogenous and exogenous compounds, in differentiated THP-1 cells, as model of human macrophages.

*Cell cultures and treatments* - Experiments were performed with THP-1 cells (ATCC TIB-202), a human myelomonocytic cell line displaying macrophage-like activity after differentiation and obtained from the American Type Culture Collection (Manassas, VA, USA). THP-1 cells were grown in RPMI medium supplemented with 10% FBS (PAA, Pasching, Austria), 2 mM L-glutamine, and a mixture of antibiotics (5 mg/mL penicillin and 5 mg/mL streptomycin; Invitrogen, Carlsbad, CA, USA). Cells were cultured at 37ºC in a humidified atmosphere containing 5% CO_2_. Unless otherwise stated, cells were seeded in 10 cm plates at a density of 1 × 10^7^ cells/well. To promote differentiation into macrophages, cells were exposed to phorbol 12-myristate 13-acetate (20 μM) for 24 h. Subsequently, cells were incubated in the presence of BZL (2, 20, and 200 μM, 48 h) as stated previously (Rigalli et al. 2012). Preliminary experiments demonstrated that 48 h was the shortest exposure time that resulted in significant protein induction. DMSO only was added to control cells (C). The medium was replaced every 24 h. The final concentration of DMSO in the culture media was always below 0.1%.

*Western blotting* - Cells were washed twice with cold phosphate buffered saline (PBS) and scraped in RIPA buffer (Thermo Scientific, Rockford, IL, USA) supplemented with PMSF (17 μg/mL), leupeptin (15 μg/mL), and pepstatin A (5 μg/mL) as protease inhibitors. Western blotting was performed as previously described (Ruiz et al. 2013).

*RNA isolation and real time reverse transcriptase polymerase chain reaction (RT-PCR)* - After cell treatment, total RNA was isolated using TRIzol® reagent (Invitrogen). cDNA was synthesised from 1 μg of total RNA using Superscript III Reverse Transcriptase (Invitrogen) and random hexamers according to the manufacturer’s instructions. Real time PCR reactions were performed on a MX3000P system (Agilent Technologies, Santa Clara, CA, USA) with Platinum Taq DNA Polymerase (Invitrogen) and SYBR Green quantification. Results for ABCB1 and ABCC2 mRNA were normalised to the expression of 18S rRNA as the housekeeping gene using the primers (1 μM) described by Rigalli et al. (2012) based on the 2^-ΔΔCt^ method (Pfaffl 2001). Stability of 18S rRNA was verified in THP-1 cells treated with BZL (200 µM, 48 h). Ct values did not show significant changes between control and BZL treated cells (control: 9.72 ± 0.26, BZL: 10.10 ± 0.25, expressed as mean ± standard deviation, n = 3, p >0.05, Student’s *t*-test). The specificity of each reaction was verified with a dissociation curve, using a temperature range of 55ºC to 95ºC with continuous fluorescence measurements.

*Functional assays* - We estimated the activity of each transporter using different experimental strategies that were found to be optimal for each case (Rigalli et al. 2012):

*P-gp activity assessed by Rh123 accumulation* - P-gp activity was assessed by flow cytometry using a Cell Sorter BD FACSAria II (Becton Dickinson, Heidelberg, Germany). Cells (1 × 10^6^/sample) were incubated (30 min, 37ºC) in the dark with the model substrate Rh123 (0.4 µM), followed by incubation (50 min, 37ºC) with the specific P-gp inhibitor PSC833 (10 µM) or with medium alone. Intracellular fluorescence was quantified using a blue laser (488 nm, 20 mW) and a FITC detection filter (530/30 nm) and analysed with FACSDiva Software (Becton Dickinson) (König et al. 2010).

*MRP2 activity assessed by DNP-SG efflux* - The activity of MRP2 was evaluated by measuring the extrusion dinitrophenyl-S-glutathione (DNP-SG) as previously reported (Rigalli et al. 2012). To confirm MRP2 participation, MK571 (10 µM) was added as an inhibitor.

*Intracellular BZL concentration* - To evaluate the possibility that BZL pre-treatment results in modification of its own intracellular concentration, the amount of BZL in cell lysates was evaluated as previously described (Rigalli et al. 2012). Briefly, THP-1 treated cells were incubated with 100 µM BZL for 2 h, in the presence or absence of PSC833 (10 µM). Cells were washed twice with cold PBS and then lysed by sonication. Because of its amphiphilic characteristics, it is assumed that BZL passively enters the cells. Retention of BZL in cells after this period was inversely correlated with its extrusion; BZL was measured in supernatant by HPLC (Rigalli et al. 2012).

*Chemicals* - BZL, 1-chloro-2,4-dinitrobenzene (CDNB), MK571, rhodamine 123 (Rh123), phenylmethylsulfonyl fluoride (PMSF), pepstatin A, and leupeptin were from Sigma-Aldrich (St. Louis, MO, USA). DMSO was purchased from Merck (Darmstadt, HE, Germany). PSC833 was from Tocris Bioscience (Bristol, QL, UK). All other chemicals were of analytical grade purity.

*Statistical analysis* - Data are presented as the mean ± standard deviation (SD). Statistical analysis was performed using a Student’s *t*-test (for comparison between two groups) or a one-way ANOVA followed by the Newman-Keuls or Dunnett’s post-hoc test, (for more than two experimental groups). Significance was set at p < 0.05. Analyses were performed using GraphPad Prism 3.0 software (GraphPad Software, La Jolla, CA, USA).

*Effect of BZL treatment on the expression of P-gp and MRP2* - THP-1 cells were selected to explore the effect of BZL on the expression and activity of human P-gp and MRP2. After differentiation and subsequent culture for 48 h in the presence of BZL, the levels of P-gp and MRP2 were quantified by western blotting using cell lysates. Fig. 1 clearly shows a concentration-dependent induction of P-gp and MRP2 (Fig. 1A-B, respectively). The concentration of 200 μM was selected for the following experiments.

**Fig. 1:**
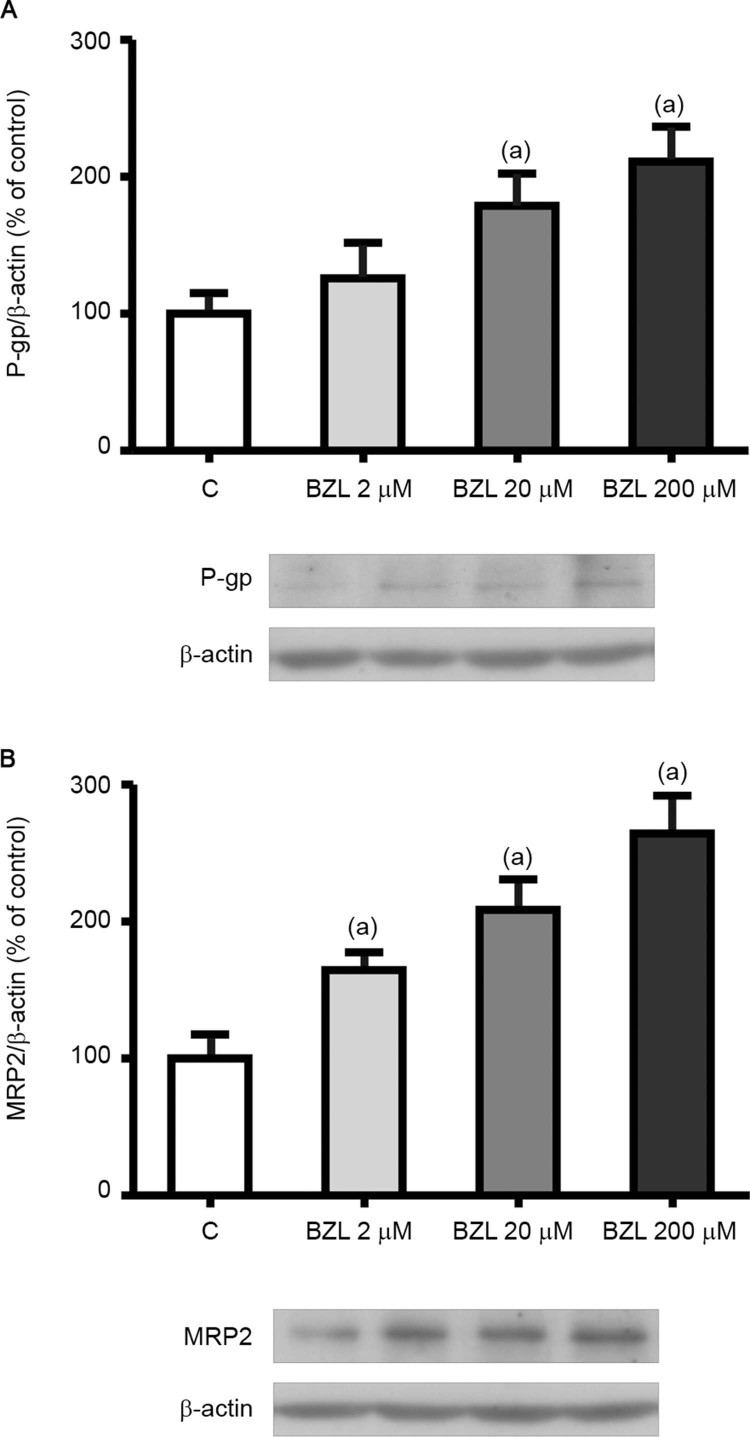
: effect of benznidazole (BZL) on transporter expression in cell lysates. P-glycoprotein (P-gp) (A) and multidrug resistance-associated protein 2 (MRP2) (B) were detected by western blotting in THP-1 total cell lysates after 48 h of treatment with BZL (2, 20, and 200 µM) or vehicle (control, C). Equal amounts of total protein (120 µg) were loaded in the gels. MRP2 and P-gp optical density (OD) was related to β-actin OD. Typical western blots are shown at the bottom. Uniformity of loading and transfer from the gel to the polyvinylidine difluoride membrane were controlled by Ponceau S staining. The data on OD (% of C) are presented as the mean ± standard deviation (SD) (n = 3; p < 0.05, one-way ANOVA followed by Dunnett’s post-hoc test). (a) Different from C.

Real time PCR results showed that compared to controls, mRNA levels of ABCB1 (C: 100 ± 25% vs BZL: 193 ± 65%) and ABCC2 (C: 100 ± 32% vs BZL: 173 ± 28%) (n = 6; p < 0.05, Student’s *t*-test) were increased in BZL-treated cells, suggesting transcriptional regulation of the respective genes.

*Effect of BZL treatment on P-gp and MRP2 activities* - To evaluate the functional impact of P-gp and MRP2 up-regulation, we estimated the activity of both transporters. We observed that increased expression of P-gp by BZL indeed correlated with reduced intracellular content of its substrate Rh123 (-10%) when compared to that in control cells (Fig. 2A). The intracellular level of Rh123 was increased by PSC833 in both control and BZL-treated cells (+10% and +16%, respectively), confirming a contribution of P-gp to Rh123 efflux.

**Fig. 2:**
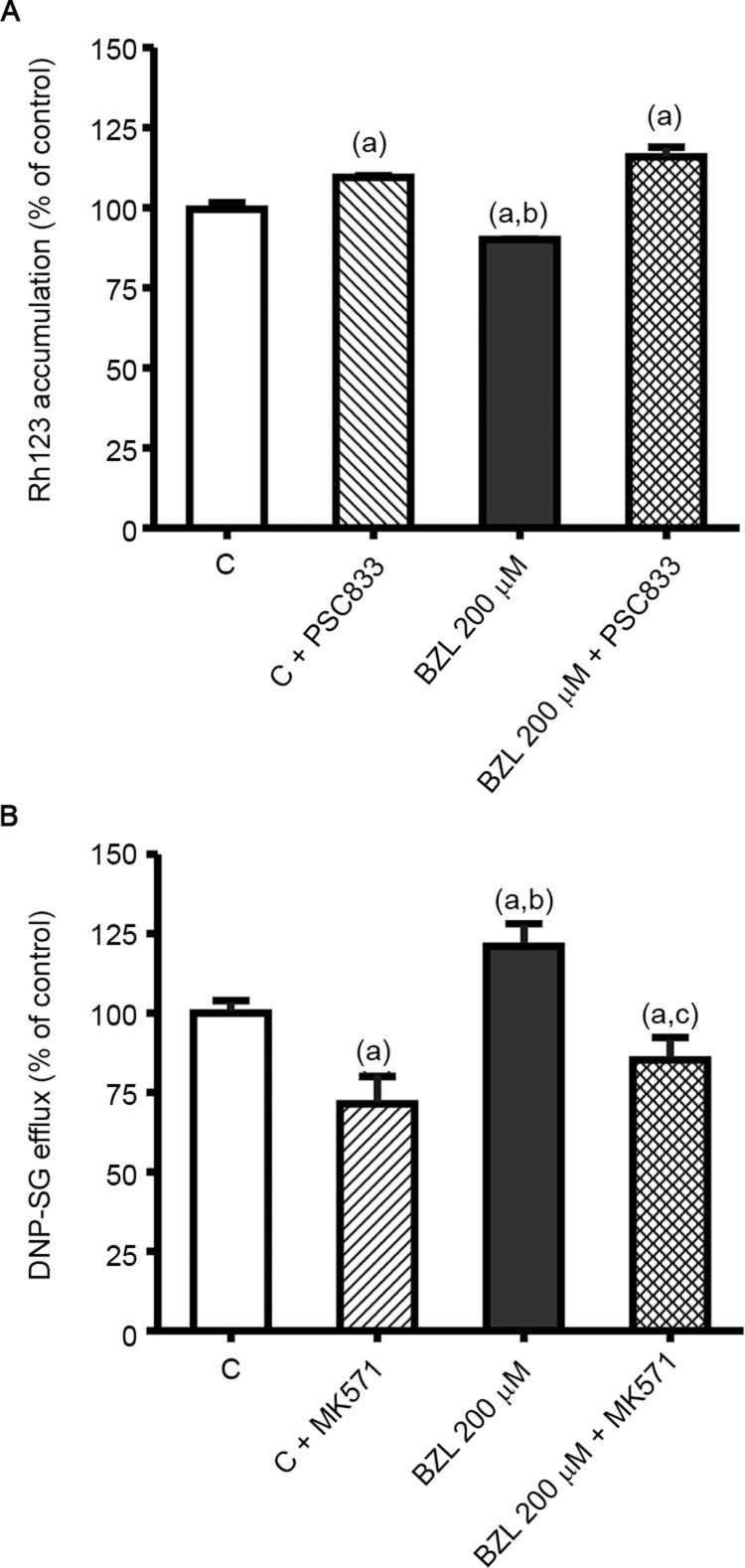
: effect of benznidazole (BZL) on transporter activity. (A) Accumulation of Rh123, in the presence or absence of PSC833 (10 µM), was inversely correlated with P-glycoprotein (P-gp) activity in cells treated with BZL (200 µM) or vehicle (control, C) for 48 h. Data are presented as percentages normalised to the accumulation of Rh123 in C, considered as 100%, and were expressed as the means ± standard deviation (SD) (n = 3-4; p < 0.05, one-say ANOVA followed by Newman-Keuls post-hoc test). (a) Significantly different from C, (b) significantly different from C+PSC833, (c) significantly different from BZL 200 µM. (B) Extrusion of DNP-SG, in the presence or absence of MK571 (10 µM), was determined in supernatants of cells treated with BZL (200 µM, 48 h) or vehicle (control, C) by HPLC, as a measure of multidrug resistance-associated protein 2 (MRP2) activity. Samples were taken after 30 min of incubation. Data (means ± SD, n = 3-4; p < 0.05, one-way ANOVA followed by Newman-Keuls post-hoc test) are presented as the percentage relative to DNP-SG extrusion in control cells. (a) Significantly different from C, (b) significantly different from C+MK571, (c) significantly different from BZL.

The excretion rate of DNP-SG in BZL-treated cells was higher (+21%) than in control cells (Fig. 2B), in agreement with the higher content of MRP2 protein. The addition of MK571 inhibited the efflux of DNP-SG both in control and BZL-treated cells (-28% and -15%, respectively), consistent with the participation of MRP2.

*Effect of BZL treatment on its intracellular concentration* - When THP-1 cells were pre-treated with BZL (200 µM, 48 h) or vehicle, and further incubated with BZL (100 µM, 2 h) for BZL transport studies, its intracellular accumulation was significantly lower in pre-treated cells (-18%), indicating a rise in drug extrusion (Fig. 3). The presence of PSC833, a specific inhibitor of P-gp, increased the intracellular BZL concentration both in pre-treated and control cells, confirming the participation of P-gp in BZL efflux.

**Fig. 3:**
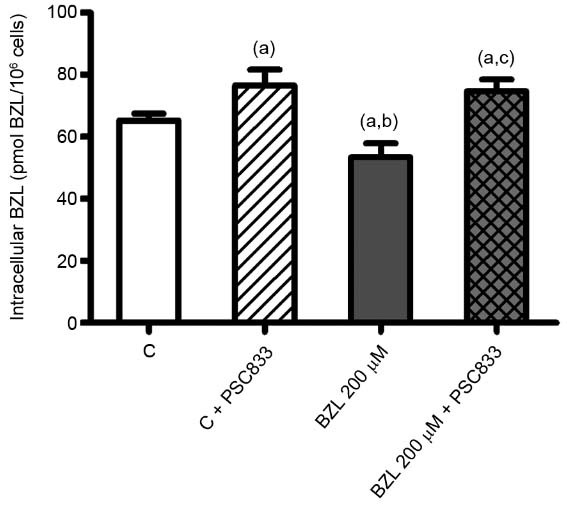
: benznidazole (BZL) intracellular concentration. THP-1 cells were pre-treated with BZL (200 µM, 48 h) or vehicle (control, C). Cells were then loaded with BZL (100 µM, 2 h) with or without PSC833 (10 µM, a specific inhibitor of P-glycoprotein (P-gp). BZL accumulation was determined in cell lysates by HPLC. Data (means ± standard deviation (SD), n = 4-5; p < 0.05, one-way ANOVA followed by Newman-Keuls post-hoc test) are expressed as pmol of BZL/106 cells. (a) Significantly different from C, (b) significantly different from C+PSC833, (c) significantly different from BZL.

In the current study, we evaluated the effect of BZL treatment, the only antichagasic drug available in most endemic countries, on the expression and activity of two important ABC proteins in a macrophage cell line. BZL was able to induce P-gp (ABCB1) and MRP2 (ABCC2) protein expression in a concentration-dependent manner. We also found that the up-regulation of P-gp and MRP2 correlated with increased activity of these transporters, suggesting that the intracellular concentration of drugs administered along with BZL, including BZL itself, could be suboptimal because of increased efflux mechanisms. ABC transporters play a crucial role in limiting the accumulation of therapeutic drugs in cells and can effectively confer resistance to a wide range of compounds. This is particularly significant for intracellular infections, in which the intracellular drug concentration can severely affect its chemotherapeutic efficacy. In this regard, it was reported that the intracellular accumulation of some antibiotics is modulated in J774 macrophages by the activity of drug efflux pumps (Seral et al. 2003a). Thus, verapamil, cyclosporine, and GF120918 (known inhibitors of P-gp) increased the accumulation of azithromycin, whereas gemfibrozil or probenecid (inhibitors of MRPs and other transporters of organic anions) increased the accumulation of ciprofloxacin (Seral et al. 2003a). Moreover, the same group demonstrated that gemfibrozil and verapamil made ciprofloxacin or azithromycin more effective against *Listeria monocytogenes* and *Staphylococcus aureus*, respectively in J774 infected macrophages (Seral et al. 2003b). Furthermore, Lemaire et al. (2007) found that daptomycin acts against phagocytized *S. aureus* in THP-1 macrophages and MDCK cells. However, its intracellular accumulation and its related activities were partially affected by P-gp modulation.

We previously reported that P-gp is involved in the extrusion of BZL in HepG2 cells (Rigalli et al. 2012). In this work, we demonstrated that the intracellular BZL concentration was lower in BZL-pre-treated THP-1 cells than in control cells suggesting that P-gp induction was responsible for the increased excretion of BZL. This was confirmed by the use of PSC833. Consequently, the co-administration of BZL with a P-gp inhibitor could mitigate treatment failure or improve the efficacy of treatment in Chagas disease.

BZL treatment (5-10 mg/kg/day, 60 days, p.o.) is recommended during the acute phase (showing up to 80% parasitological cure), and recent chronic infections (Andrade et al. 2011). However, the treatment of chronically infected adult patients, representing the most prevalent form of the disease, is still controversial because drug efficacy declines with the advancement of infection (Gaspar et al. 2015). In our study, up-regulation of P-gp and MRP2 transporters in THP-1 cells treated with BZL occurred in a concentration-dependent manner. However, it is not known if this could be extrapolated to patients treated with BZL. Indeed, our study shows only slight variations in the transporter expression and activity. However, it is well known that transporter induction by xenobiotics might vary within the population (Jigorel et al. 2006). Thus, a higher induction in vivo contributing to therapeutic failure cannot be ruled out. The typical doses used for the treatment of Chagas disease lead to plasma concentrations up to 110 µM; however, higher BZL plasma levels can be reached in patients subjected to cardiac transplantation, since higher doses of BZL are used for this application (Pedrosa et al. 2001, Soy et al. 2015). In the current study we observed a mild up-regulation of ABC transporters in vitro after a shorter exposure time (48 h), but a strong response could be expected after longer exposure time. Nevertheless, extrapolation of the results to an in vivo situation should be done with caution, since the treatment of patients can last up 90 days in cases of reactivation of the disease (Andrade et al. 2011). In addition, it is important to consider the host immunological response during *T. cruzi* infection and diet and hormonal status, among other factors, which could also modulate ABC transporter expression and activity.

In conclusion, we reported an increase in expression and activity of P-gp and MRP2 in THP-1 cells treated with BZL with potential consequences in the pharmacodynamics of co-administered drugs including BZL itself.
